# Randomised Double-Blind Placebo-Controlled Trial of Inulin with Metronidazole in Non-Alcoholic Fatty Liver Disease (NAFLD)

**DOI:** 10.3390/nu12040937

**Published:** 2020-03-27

**Authors:** Clara Yieh Lin Chong, David Orr, Lindsay D. Plank, Tommi Vatanen, Justin M. O’Sullivan, Rinki Murphy

**Affiliations:** 1 Liggins Institute, The University of Auckland, Auckland 1142, New Zealand; clara.chong@auckland.ac.nz (C.Y.L.C.); t.vatanen@auckland.ac.nz (T.V.); justin.osullivan@auckland.ac.nz (J.M.O.); 2 New Zealand Liver Transplant Unit, Auckland City Hospital, Auckland 1023, New Zealand; 3 Department of Surgery, Faculty of Medical and Health Sciences, The University of Auckland, Auckland 1142, New Zealand; l.plank@auckland.ac.nz; 4 Department of Medicine, Faculty of Medical and Health Sciences, The University of Auckland, Auckland 1142, New Zealand

**Keywords:** prebiotics, alanine aminotransferase, antibiotic, Optifast, gut microbiome, inulin, metronidazole

## Abstract

*Background*: Non-alcoholic fatty liver disease (NAFLD) can be ameliorated by weight loss although difficult to maintain. Emerging evidence indicates that prebiotics and antibiotics improve NAFLD. *Aim*: To determine whether inulin supplementation after brief metronidazole therapy is effective in reducing alanine aminotransferase (ALT) and maintaining weight loss achieved through a very-low-calorie diet (VLCD) among people with NAFLD. *Methods*: Sixty-two people with NAFLD commenced 4-week VLCD using Optifast meal replacements (600 kcal/day). Sixty were then randomised into a 12-week double-blind, placebo-controlled, parallel three-arm trial: (1) 400 mg metronidazole twice daily in Week 1 then inulin 4 g twice daily OR (2) placebo twice daily in week one then inulin OR (3) placebo-placebo. Main outcomes were ALT and body weight at 12 weeks. Fecal microbiota changes were also evaluated. *Results*: Mean body mass index (BMI) and ALT reduced after VLCD by 2.4 kg/m^2^ and 11 U/L, respectively. ALT further decreased after metronidazole-inulin compared to after placebo-placebo (mean ALT change −19.6 vs. −0.2 U/L, respectively; *p* = 0.026); however, weight loss maintenance did not differ. VLCD treatment decreased the ratio of *Firmicutes*/*Bacteroidetes* (*p* = 0.002). *Conclusion*: Brief metronidazole followed by inulin supplementation can reduce ALT beyond that achieved after VLCD in patients with NAFLD.

## 1. Introduction

Non-alcoholic fatty liver disease (NAFLD) is defined by the pathological accumulation of fat in the liver and is now the leading cause of chronic liver disease [1]. NAFLD encompasses a spectrum of diseases ranging from simple fatty liver (steatosis) through to non-alcoholic steatohepatitis (NASH), which, in turn, leads to fibrosis, irreversible cirrhosis and, finally, hepatocellular carcinoma (HCC) in a small proportion of people [2,3]. The milder simple steatosis is characterised by the ectopic accumulation of fat in the liver, usually associated with energy-surplus-induced obesity. It is believed that multiple parallel factors (diet, insulin resistance, mitochondrial dysfunction and inflammation), acting synergistically in genetically predisposed individuals, are implicated in the development and progression of NAFLD.

An accumulating number of animal and human studies suggest a compelling role for gut microbiota in NAFLD, which is both transmitted by gut microbiota and reversed by a combination of ciprofloxacin and metronidazole antibiotics in animal models [4]. NAFLD is associated with dysbiosis of the gut microbiota, which is thought to lead to increased gut permeability, and abnormal choline and bile acid metabolism, leading to inflammation and increased hepatic fat accumulation [5]. An indication of the involvement of gut microbiota in NAFLD development was first apparent when hepatic steatosis developed in patients undergoing jejunal–ileal bypass surgery, coinciding with intestinal bacterial overgrowth in the blind loop. The hepatic steatosis regressed once patients were treated with the antibiotic metronidazole [6] which is commonly used for the treatment of small intestinal bacterial overgrowth [7]. While small intestinal bacterial overgrowth has been shown to be more prevalent in NAFLD [8,9,10,11], antibiotic treatment of NAFLD has not been investigated due to concerns about long-term use being associated with side effects, antimicrobial resistance and uncertain efficacy.

The cornerstone of NAFLD treatment currently is to offer lifestyle advice that targets 7% to 10% weight loss and is proven to be effective [12,13]. Recent evidence shows that very-low-calorie diets (VLCDs) [14] and bariatric surgery [15] are very effective in achieving weight loss and remission of associated comorbidities. Both these strategies alter gut microbiota, but to a lesser extent after dietary modification than after surgery [16,17,18]. However, the maintenance of weight loss remains a challenge and better alternatives to targeting specific mechanistic dysfunction are needed.

Prebiotics, which are nondigestible food ingredients that are fermented in the gut and modulate microbiota in a favourable way for the host, have shown promise in the treatment of NAFLD. A systematic review of 26 randomised controlled trials investigating the metabolic benefits of prebiotics concluded that prebiotics improve satiety, postprandial glucose and insulin in both healthy and obese individuals [19]. A meta-analysis of nine randomised controlled trials in NAFLD showed a reduction in body mass index (BMI) and an overall improvement in aminotransferase (ALT) with the use of prebiotics [20]. However, the use of a combination of strategies targeting gut microbiota dysbiosis of NAFLD such as VLCD, metronidazole and prebiotics in succession has not previously been investigated. 

We hypothesised that the beneficial metabolic effects of short-term VLCD among adults with NAFLD could be enhanced by the brief use of metronidazole to target dysbiotic gut microbiota followed by a period of inulin supplementation to maintain this. We conducted a single centre, randomised, placebo-controlled, double-blind clinical three-arm trial of 12 weeks of inulin supplementation with or without an initial week of metronidazole cotreatment among adults with NAFLD who had all received four weeks of VLCDs.

## 2. Patients and Methods 

### 2.1. Study Design 

This study focused on adults with an established diagnosis of NAFLD attending Auckland City Hospital hepatology outpatient clinic. Patients either had histological evidence of NAFLD based on a liver biopsy, a phenotypic diagnosis based on the presence of BMI > 27 kg/m^2^ and type 2 diabetes or metabolic syndrome (WHO criteria) with an elevated ALT (male > 40 U/L, female > 30 U/L) and age >18 years and <75 years. Exclusion criteria were alcohol consumption of more than 20 g per day for at least 3 consecutive months during the previous 5 years as assessed by a questionnaire. Participants were excluded if they had cirrhosis, hepatitis C or another liver disease, if they were awaiting or had previous bariatric surgery, had an allergy to eggs, nuts or metronidazole, a history of drug and alcohol abuse, a calculated eGFR less than 60 mL/min (MDRD formula) or current participation in other therapeutic trials. Ethics approval was from Health and Disability Ethics Committee NTX/12/05/040/AM02; ANZCTR registration number: 12613001002774, prospectively registered on 10 September 2013. 

### 2.2. Randomisation and Treatment Groups

Sixty-two participants with NAFLD who met all eligibility criteria and provided written informed consent were provided with 3 Optifast meal replacements (600 kcal/day) per day for 4 weeks to initiate weight loss after which the 60 participants who attended the second study visit were randomly assigned to one of three parallel groups (1:1:1; Figure A1). The metronidazole and inulin group (Group MI) received metronidazole (dose of 400 mg twice daily for 7 days) along with inulin (at a dose of 4 g twice daily for 12 weeks); the placebo and inulin group (Group PI) received metronidazole-like placebo (twice daily for 7 days) along with inulin (at a dose of 4 g twice daily for 12 weeks); the placebo and inulin placebo group (Group PP) received metronidazole-like placebo (twice daily for 7 days) along with inulin-like placebo (containing maltodextrin at a dose of 4 g twice daily for 12 weeks).

The inulin dose was selected on the basis of previous prebiotic studies and was provided by Cargill Belgium. A metronidazole dose of 400 mg twice daily was selected as slightly lower than the standard dose of 400 mg three times daily used for various medical conditions, such as bacterial vaginosis, dental abscess and giardiasis, for increased adherence than three times daily. Metronidazole and matching placebo-containing maltodextrin were encapsulated by the Auckland Hospital Clinical Trials’ Pharmacy department. All participants, their health care providers and assessment staff were blinded to treatment allocation. Participants were asked to take the inulin/matching placebo powder twice daily before breakfast and before dinner using a 4 g measuring spoon and two level spoonfuls dissolved into water. All participants were given a standardised set of recommendations about lifestyle changes and diet following the initial expected weight loss period during VLCD at time of study randomisation 

**Figure 1:**
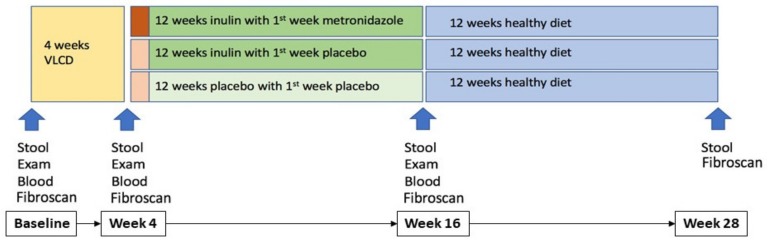
Assessment and sample collection timeline.

In total, there were four time points in this study: baseline (study entry), Week 4 (after 4 weeks VLCD pre-randomisation), Week 16 (post-randomisation, at the end of treatment) and Week 28 (post-treatment follow up phase to evaluate whether there were any persistent effects detected beyond the treatment period) as shown in Figure 1. All participants underwent assessment for body weight, height, waist and hip circumference at each of these 4 timepoints. Blood samples for assessment of fasting lipids, glucose, insulin and liver Fibroscan CAP were obtained at baseline, Week 4 and then Week 16. 

### 2.3. Stool Sample Collection 

Stool samples were collected at each time point (Figure 1): baseline, Week 4, Week 16 and Week 28. Study participants collected the stool samples at home, using a sterile collection tube, prior to their hospital visits. Stool samples were stored at −70 °C from the beginning of the study (2013/2015) until DNA extraction was performed (2017).

DNA was extracted from stool samples using the QIAamp^®^ Fast DNA Stool Mini Kit according to the manufacturer’s protocol. Extracted DNA quality and quantity were measured using a NanoPhotometer N60 (IMPLEN, Germany; Table S1) and a Qubit (Invitrogen, US).

### 2.4. 16S rRNA Gene Amplicon Sequencing

Extracted DNA (mean yield = 6733.4 ng; mean 260/280 = 1.97; mean concentration = 33.7 ng/µL) was sent to the School of Biological Sciences (The University of Auckland, New Zealand) for 16S rRNA amplicon sequencing on an Illumina MiSeq sequencing platform. Sequences are available from SRA project number SUB5068044. Then, 16S rRNA gene amplicon sequencing (16S sequencing) libraries were prepared using the Nextera XT kit (Illumina). V3 and V4 regions were targeted for 16S sequencing by using the 16S Amplicon PCR Forward Primer (TCGTCGGCAGCGTCAGATGTGTATAAGAGACAGCCTACGGGNGGCWGCAG) and Reverse Primer (GTCTCGTGGGCTCGGAGATGTGTATAAGAGACAGGACTACHVGGGTATCTAATCC). 

All amplicons were sequenced on the Illumina MiSeq 600 cycle run to generate an average of 121,346 sequence reads with paired-end (300 bp each) reads per sample. 

### 2.5. 16S rRNA Amplicon Sequence Analyses

The 16S sequencing data were processed using QIIME 2 (v. 2018.4) [21]. Briefly, sequence quality control and denoising were performed using DADA2 [22]. The quality control step also included the filtering of PhiX reads and chimeric sequences. The sequences obtained after denoising were then classified using Greengenes 13_8 release data to identify amplicon sequencing variants (ASVs) for sequences with >99% sequence similarity. Samples that were included in downstream analyses had filtered sequence counts ranging from 12,123 to 109,977 (median 53,071). Three samples with less than 10,000 sequencing reads were removed.

### 2.6. Primary and Secondary Outcomes

The primary outcome was the proportion maintaining a ≥7% weight loss at the end of the 12-week variable treatment period compared to their baseline (before the fixed 4-week VLCD treatment period). Secondary outcomes measured at Week 16 (the end of the 12-week variable treatment period) included changes in ALT, glycaemia, lipids, Fibroscan^®^ CAP from what was achieved at Week 4 (after VLCD treatment period) and the changes in gut microbial community from baseline to 28 weeks.

### 2.7. Statistical Analysis

The planned sample size for this pilot study was 60 subjects with an equal assignment to each of the three study groups (20 per group). We estimated that, with this sample size, the study would have 80% power to detect a difference in the proportion achieving a sustained weight loss of ≥7% at the end of the 12-week treatment period which we anticipated would be achieved by 50% of those receiving metronidazole and inulin supplemented diet, compared to 5% in the other two placebo-containing groups, with a two-sided type 1 error of 0.05. The primary outcome was assessed using Fisher’s exact test. Pre-planned analyses for secondary outcomes were comparisons of the changes over the 12-week variable treatment period in the MI and PI groups with those in the PP group. Two-sample *t*-tests were used for these comparisons for normally distributed data and Mann–Whitney *U*-tests for non-normal data. Within-cohort changes over the VLCD period were analysed using paired *t*-test or Wilcoxon signed-rank test as appropriate. Data are presented as mean (SD) or median (Quartile 1, Quartile 3) for normally and non-normally distributed data, respectively.

### 2.8. Statistical Methods for Microbiota Analysis

Omnibus associations between microbial community structure and patient metadata were assessed using Permutational Analysis of Variance (PERMANOVA) (adonis function from the vegan package in R, 10,000 permutations) and Bray–Curtis dissimilarities. Wilcoxon matched-pairs signed-rank tests were used to assess relative abundance across timepoints. Associations between individual microbial taxa and patients’ metadata were assessed using Multivariate Association with Linear Models (MaAsLin) [23], controlling for age as a possible confounding factor and repeated sampling per individual by a random effect. The Kruskal–Wallis test was used when more than two independent groups were compared. ASVs that were present in less than 20% of samples were filtered out. *p*-values were corrected for multiple testing using the Benjamini–Hochberg procedure [24,25] and FDR corrected *p*-values (*q*-values) were reported.

## 3. Results

### 3.1. Participants

**Figure A1:**
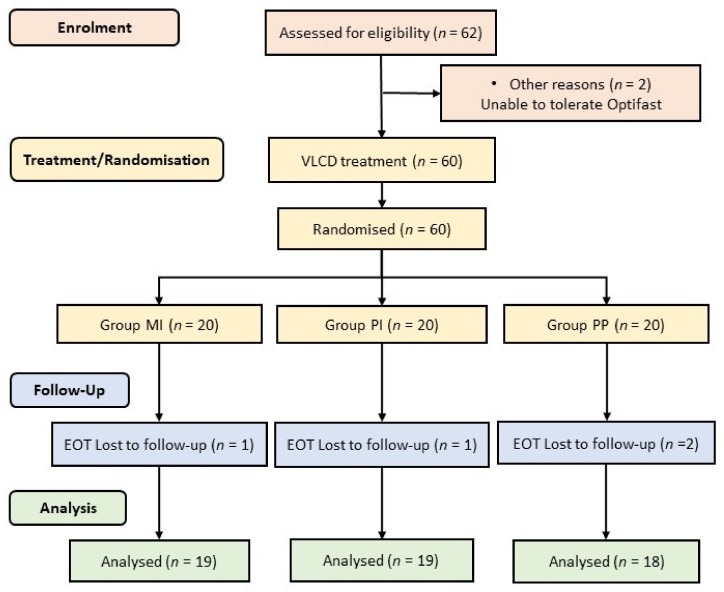
Flow diagram showing different phases of the study and number of participants at each stage. EOT: end of treatment; MI: metronidazole-inulin; PI: placebo-inulin; PP: placebo-placebo.

Enrollment into this trial occurred between March 2013 and March 2015. Sixty-two participants entered the study and began VLCD, of whom 60 attended Visit 2 and were then randomised: 20 attended metronidazole and inulin (MI), 20 attended metronidazole placebo and inulin (PI) and 20 attended metronidazole placebo and inulin placebo (PP). Participant flow through the trial is shown in Figure A1. The mean age was 50 years (range 19–71), BMI 31.6 kg/m^2^ (range 25.2–41.9) and ALT 66 U/L (range 30–141). The three groups were well matched with respect to demographic characteristics, clinical and laboratory data at study entry and after four weeks of VLCD (Table A1). Over the VLCD period, there were significant reductions in body weight, waist:hip ratio, blood pressure, ALT and gamma-glutamyl transferase (GGT), total and LDL cholesterol, triglycerides, fasting glucose, HbA1c, CRP and Fibroscan CAP score. 

### 3.2. Primary and Secondary Outcomes

Of the 62 participants who were assessed at baseline, 60 were randomised and 56/60 (93.3%) participants completed the study. The clinical endpoint of achieving sustained weight loss of ≥7% at 16 weeks compared to baseline pre-VLCD was reached by 55% in group MI compared with 53% in group PI and 35% in group PP. These were not statistically significantly different (*p* = 0.473). At 28 weeks, a sustained weight loss of ≥7% was reached by 42% in group MI, 35% in group PI and 25% in group PP (*p* = 0.584).

Although there was no difference in BMI between the three treatment groups at 12 weeks, only the group receiving inulin with an initial one week of metronidazole, group MI, had a significant, further improvement in ALT (Table A2). However, this group had no significant change in Fibroscan^®^ CAP score or in other markers of metabolic syndrome such as blood pressure, fasting lipids and glycaemia. No cases of adverse events requiring discontinuation of inulin were reported.

### 3.3. Gut Microbial Changes in Our Study Cohort

**Figure A2:**
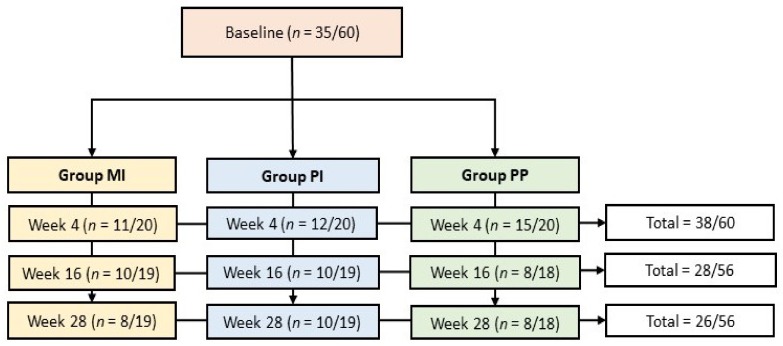
Flow diagram showing total stool sample collection at different time points from different groups. MI: metronidazole-inulin; PI: placebo-inulin; PP: placebo-placebo.

A total of 127 stool samples were obtained for analysis (Figure A2). Patient compliance in providing stool samples was highest at Week 4 with 38 (29.9%) stool samples and lowest at Week 28 with 26 (20.5%) stool samples (Figure A2). All four stool samples were obtained from 10 study participants. 

**Figure 2:**
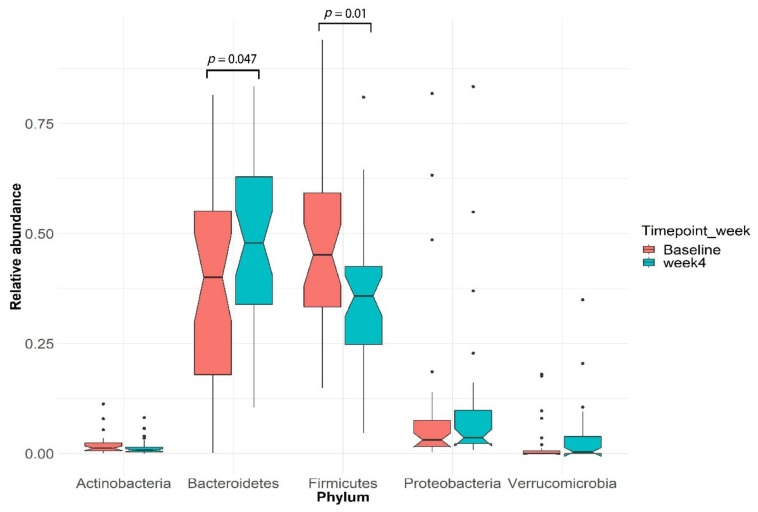
*Firmicutes* and *Bacteroidetes* are the dominant phyla in the subjects before and after very-low-calorie diet (VLCD) treatment. The figure shows boxplots of five typical human microbiota phyla. The boxes indicate the interquartile range (IQR) while the notch region shows the 95% confidence interval for the median and the whiskers extending from the boxes represent the distribution within 1.5 × IQR, with points beyond this range shown as outliers.

**Figure 3:**
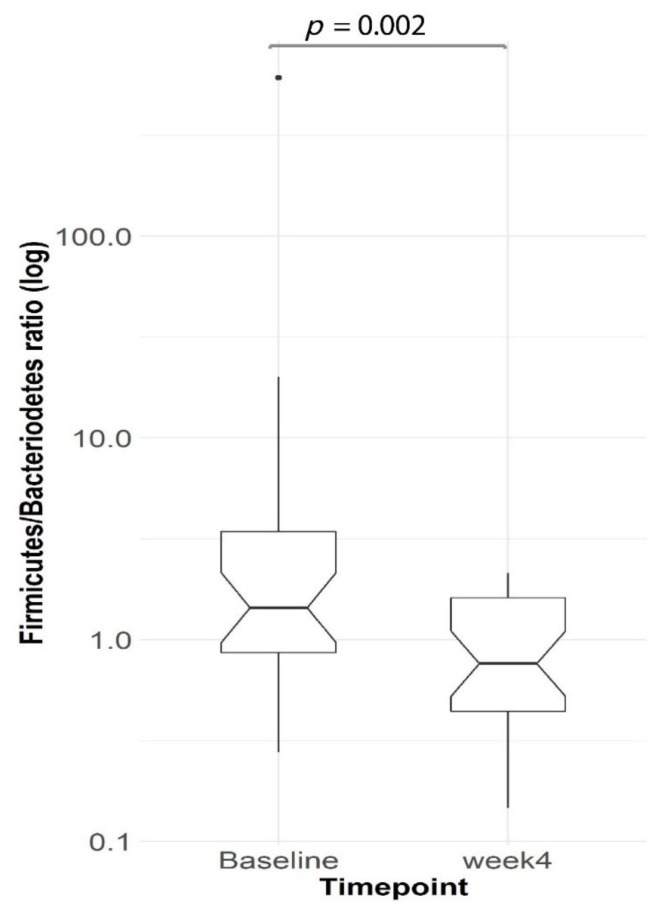
The ratio of *Firmicutes*/*Bacteroidetes* phyla decreased (Wilcoxon signed-rank test, *p* = 0.002, *n* = 30) from baseline to Week 4 after the VLCD diet. Boxplots as in Figure 2.

After Optifast VLCD: VLCD treatment explained 5.3% (PERMANOVA, *p* = 0.0024) of the variance in microbial profiles. *Bacteroidetes* and *Firmicutes* were the two most highly represented bacterial phyla in our cohort (Figure 2). After four weeks of VLCD, the relative abundance of *Bacteroidetes* increased (Wilcoxon signed-rank test, *p* = 0.047) while *Firmicutes* decreased (Wilcoxon signed-rank test, *p* = 0.01) (Figure 2). Furthermore, the ratio of *Firmicutes*/*Bacteroidetes* decreased significantly after VLCD treatment (Wilcoxon signed-rank test, *p* = 0.002, *n* = 30) (Figure 3).

**Figure 4:**
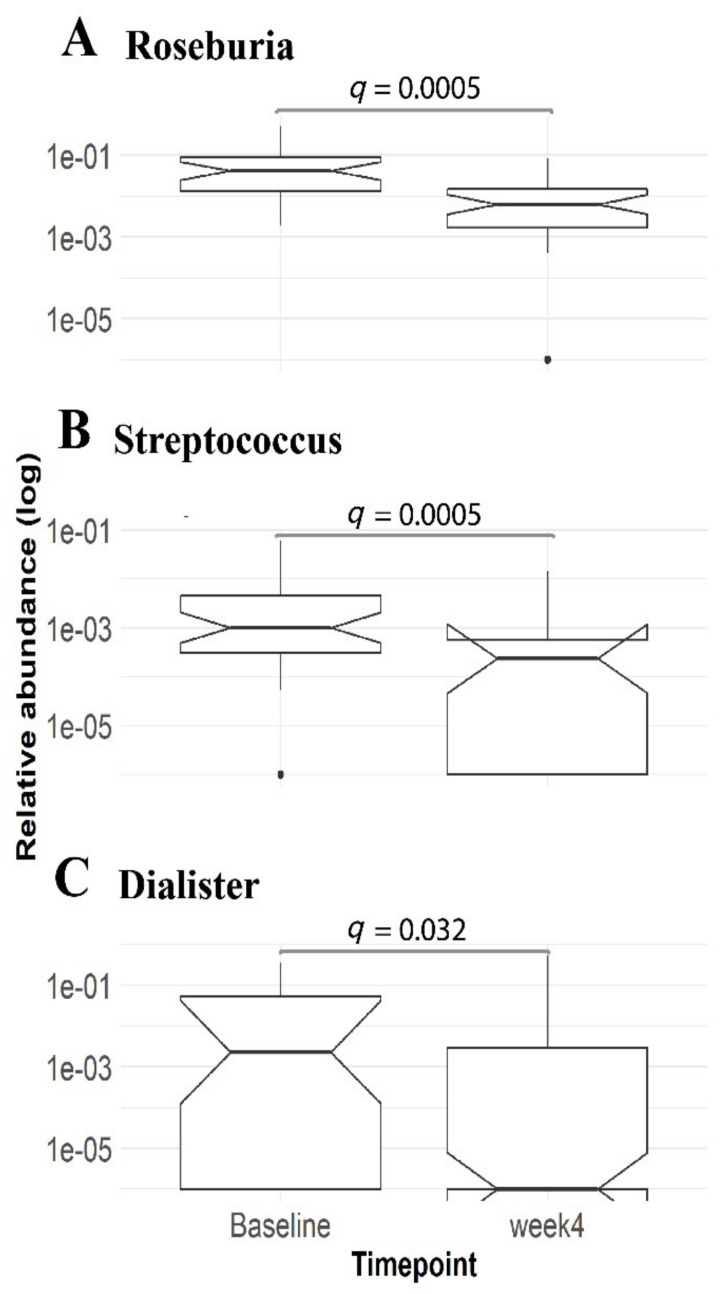
The relative abundance of genera *Roseburia* (**A**), *Streptococcus* (**B**) and *Dialister* (**C**) were lower (linear mixed-effects model, *q*-value = 0.0005, 0.0005 and 0.03, respectively) after VLCD treatment compared to baseline. Baseline, *n* = 35; Week 4, *n* = 38. Boxplots as in Figure 2.

Linear modelling identified three statistically significant genera (*q* < 0.1, Table S2), all belonging to the phylum Firmicutes. *Roseburia*, *Streptococcus* and *Dialister* genera displayed an association with the VLCD treatment and were significantly lower after VLCD treatment compared to the other time points (Figure 4). However, the microbial alpha diversity metrics showed no significant change from baseline following a VLCD diet (Shannon, *p* = 0.968; Wilcoxon signed-rank test, *n* = 30).

### 3.4. Enrichment of Distinct Gut Microbial Profile in Our Study Cohort

There were no significant differences in alpha diversity (Shannon’s diversity index) at Week 16 between intervention groups (*p* = 0.755, Kruskal–Wallis test). Similarly, comparison between Week 4 and Week 16 found no significant difference between groups (*p* = 0.949, Kruskal–Wallis test).

Linear modelling identified three taxa, genera *Roseburia*, *Anaerotruncus* and family *Lachnospiraceae*, all belonging to the phylum Firmicute, were associated to the antibiotic/prebiotic treatment period (*q* = 0.026) (Table S3). However, comparison between groups found no differences (*p* = 0.097, Kruskal–Wallis test). 

Linear modelling revealed a suggestive association between genus *Turicibacter* and plasma ALT levels (*q*-value = 0.086, Table S4) when corrected for patient age. However, neither linear modelling nor PERMANOVA identified any significant associations between microbial taxa and plasma ALT in the cross-sectional model at Week 16 (*n* = 28 samples).

## 4. Discussion

There are currently few approved treatment options for NAFLD patients beyond dietary measures to lose weight. In this study, four weeks of VLCD resulted in a significant weight loss. Subsequent inulin supplementation for 12 weeks, with or without an initial one week of metronidazole), did not improve weight loss maintenance. The transition to a real food diet after a period of VLCD meal replacements is usually associated with weight regain and recurrence of NAFLD. Indeed, only 48% of participants were able to maintain a ≥7% weight loss after 12 weeks of transition to a real food diet. Despite similar weight loss maintenance, the group who received metronidazole and inulin (after the initial VLCD period) achieved a further significant reduction in ALT. The reduction in ALT suggests reduced steatohepatitis, which, surprisingly, in our study—in contrast to other prebiotic studies that have shown an improvement in ALT commensurate with weight loss [20]—occurred without further weight loss. This finding supports the potential role of metronidazole in improving steatohepatitis through the treatment of intestinal bacterial overgrowth and/or through altering gut microbial functions that enhance efflux of free fatty acids and de novo lipogenesis in the liver. These mechanisms can occur without weight loss as the cause and are not necessarily apparent by simple characterisation of microbial abundances in the faeces. This is because there is substantial inter-individual variability in gut microbiota among patients with NAFLD and bacterial abundance in faecal samples do not directly demonstrate activity or metabolite production of the taxa present in the small intestine.

Nonetheless, four weeks of VLCD (Optifast) had a major effect on decreasing the ratio of *Firmicutes*/*Bacteroidetes* in faeces, as well as decreasing the abundance of genera *Roseburia*, *Streptococcus* and *Dialister*. The genus *Roseburia*, a member of clostridial cluster XIVa [26], consists of obligate Gram-positive anaerobic bacteria and is an important butyrate-producing colonic bacterium [27,28,29,30] and suggested to be able to alleviate inflammation by stimulating Treg cell differentiation [31,32]. Butyrate is a short-chain fatty acid produced mainly by the enteric microbiome [33,34]. It is a crucial element in the normal development of colonic epithelial cells [35] and preferred energy source in the colonic mucosa [36]. A previous study has shown that a butyrate-producing probiotic MIYAIRI 588 strain of *Clostridium butyricum* effectively improved hepatic indexes in an animal model [33]. Butyrate has also been suggested to confer various beneficial metabolic effects such as enhancing mitochondrial activity [34], increasing insulin sensitivity [37], conveying anti-inflammatory potential [38] as well as increasing intestinal barrier function [39]. However, the role of butyrate in NAFLD is controversial as patients with NASH were shown to have higher faecal butyrate compared to healthy subjects [40]. *Roseburia* has also been detected as significantly elevated in NAFLD patients compared to healthy controls [41]. This genus is suggested to be one of the gut microbiota biomarkers that is shared by obese patients with metabolic disease and is negatively associated with body mass index (BMI) [32]. Ironically, a depletion of *Roseburia* was observed after four weeks of Optifast VLCD treatment, with a significant reduction in BMI in our study. In fact, our observation is similar to Duncan et al. [42] and, subsequently, Alemán et al. [43] who observed a significant reduction of genus *Roseburia* after VLCD intervention. Since *Roseburia* are predominantly polysaccharide-degrading bacteria [44], we postulate that the observed reduction in the genus *Roseburia* is actually due to reduced dietary carbohydrates from VLCD and not directly linked to BMI. 

Similarly, the genus *Streptococcus*, a possible biomarker of NAFLD [45] reduced significantly after VLCD treatment compared to baseline (Figure 4). This corroborates previous work where *Streptococcus* was enriched in NAFLD and NAFLD-cirrhosis patients [46,47] compared to both healthy subjects [48,49] and obese individuals [45]. Future studies could investigate metabolic activities and molecular mechanisms linking *Streptococcus* and NAFLD aetiology.

Optifast-based VLCD reduced the alpha diversity (Shannon’s diversity index) of gut microbiota seen between baseline to Week 4 (Figure S1). Altered diversity has also been shown to occur in a similar Optifast-based VLCD study of three months duration in 18 obese participants, although changes regressed during the subsequent weight maintenance phase and return to a real food diet [18]. It is noticeable that upon transition to a food diet, metronidazole-inulin and placebo-inulin groups both shared a similar fluctuation pattern in Shannon’s diversity index for all time points compared to the placebo-placebo group which demonstrated a relatively stable pattern.

Due to limited stool sample collection after VLCD, lack of statistical power precluded the assessment of any gut microbial differences in abundance between metronidazole treated and non-metronidazole treated groups. Further, we cannot rule out the possibility that low compliance in the stool sample collection may have added further bias in microbiome analysis. However, linear modelling revealed that the genus *Turicibacter* was associated with the plasma ALT levels within placebo-inulin and placebo-placebo groups. *Turicibacter* has been suggested to be responsive to the cholesterol level in the diet [50]. This is in line with the fact that hepatic free cholesterol accumulation and altered cholesterol homeostasis will lead to liver injury and eventually contribute to the pathogenesis of NAFLD/NASH [51]. We suggest an association between *Turicibacter* and plasma ALT levels which clearly need further research. 

Finally, maltodextrin may not have been as inert as a placebo should be, given there is some evidence that maltodextrin detrimentally impacts the intestinal environment [52,53,54]. However, most of these studies used much higher doses of maltodextrin than was used in the form of placebo to match inulin in this study.

## 5. Conclusions

In conclusion, this is the first clinical trial evidence that supplementation with prebiotic inulin following brief metronidazole therapy can further reduce ALT after four weeks of VLCD therapy in patients with NAFLD. A prominent shift in phyla Firmicutes, Bacteroidetes, genera *Roseburia*, *Streptococcus* and *Dialister* were seen after four weeks of Optifast treatment. Unfortunately, limited stool samples collection after VLCD treatment resulted in insufficient power to detect a significant difference in gut microbiota with additional metronidazole or inulin. Nevertheless, a potential role of metronidazole, together with inulin in altering the gut microbial function (e.g., metabolites production), is suggested to alleviate steatohepatitis, as evidenced by the reduction of ALT in the MI group. Future studies are recommended to examine the effect on microbial metabolites which does not manifest in measuring the diversity of microbiota. Furthermore, this clinical therapeutic approach requires validation in larger clinical studies with the possibility of low compliance on stool samples collection is accommodated. 

## Supplementary Materials

The following are available online at https://www.mdpi.com/2072-6643/12/4/937/s1, Figure S1: Violin plot on the Shannon’s diversity index for each group on each time point, Table S1: DNA quality and quantity measured using a NanoPhotometer N60, Table S2: Linear modelling results showing genera associated with the VLCD treatment, Table S3: Linear modelling results showing genera associated with the antibiotic/prebiotic treatment period, Table S4: Linear modelling results showing suggestive association between genus *Turicibacter* and plasma ALT levels.

## Abbreviations

VLCD	Very low calorie diet
NAFLD	Non-alcoholic fatty liver disease
ALT	Alanine aminotransferase
ASVs	Amplicon Sequence Variants

## Appendix A

**Table A1:** Baseline and week 4 characteristics of study participants.

Clinical Characteristics	Metronidazole-Inulin (*n* = 20)	Placebo-Inulin (*n* = 20)	Placebo-Placebo (*n* = 20)	Total (*n* = 60)
Age (y)	50.6 (10.4)	51.5 (13.5)	46.7 (11.2)	49.6 (11.8)
Sex (M:F)	10:10	8:12	13:7	31:29
Body mass index (kg/m^2^)				
Baseline Week 4	31.4 (3.4) 29.0 (3.4)	30.9 (3.5) 27.1 (7.3)	32.6 (4.3) 29.9 (3.8)	31.6 (3.8) 29.1 (3.6) ***
Weight (kg)				
Baseline Week 4	89.8 (12.4) 83.0 (12.0)	85.6 (15.5) 73.7 (21.6)	94.2 (15.9) 86.4 (13.4)	89.9 (14.9) 82.7 (13.3) ***
Waist:hip ratio				
Baseline Week 4	0.98 (0.07) 0.93 (0.06)	0.99 (0.04) 0.94 (0.05)	0.98 (0.06) 0.92 (0.08)	0.98 (0.05) 0.96 (0.06) ***
Blood pressure (mm Hg)				
Systolic at Baseline Systolic at Week 4 Diastolic at Baseline Diastolic at Week 4	132 (16) 121 (13) 83 (10) 76 (10)	129 (14) 121 (16) 77 (10) 74 (10)	125 (17) 121 (21) 77 (11) 74 (14)	129 (16) 121 (17) *** 79 (11) 75 (11) ***
ALT (U/L)				
Baseline Week 4	64.0 (28.6) 59.8 (25.0)	70.0 (26.1) 53.3 (22.0)	60.2 (28.1) 46.2 (21.1)	64.7 (27.4) 53.2 (23.1) **
AST (U/L)				
Baseline Week 4	34 (25, 46) 41 (31, 55)	41 (31, 47) 36 (30, 47)	33 (27, 40) 30 (27, 37)	36 (29, 45) 36(29, 43)
GGT (U/L)				
Baseline Week 4	101 (61, 141) 49 (29, 73)	164 (76, 246) 83 (35, 124)	71 (42, 134) 35 (22, 65)	93 (57, 163) 46(27, 88) ***
Bilirubin (mol/L)				
Baseline Week 4	9.5 (7.5, 13.0) 11.0 (8.5,12.0)	9.5 (6.5, 3.5) 10.0 (6.0,15.0)	9.5 (7.5, 12.0) 11.0 (8.0,15.0)	9.5 (7, 13) 10 (8, 14) *
Albumin (g/L)				
Baseline Week 4	46.0 (2.5) 45.5 (2.7)	47.4 (2.8) 47.3 (2.6)	45.3 (2.1) 46.1 (2.2)	46.2 (2.6) 46.3 (2.6)
Ferritin (μg/L)				
Baseline Week 4	228 (172) 295 (255)	214 (190) 221 (166)	254 (222) 241 (204)	232 (194) 251(208)
Cholesterol (mmol/L)				
Total (Baseline) Total (Week 4) High-density (Baseline) High-density (Week 4) Low-density (Baseline) Low–density (Week 4)	4.77 (0.98) 3.77 (1.05) 1.35 (0.43) 1.32 (0.40) 2.54 (0.74) 1.85 (0.72)	5.16 (1.29) 3.78 (1.00) 1.41 (0.36) 1.37 (0.32) 2.88 (1.28) 1.94 (0.91)	5.01 (0.86) 3.64 (0.76) 1.26 (0.27 1.18 (0.21) 2.96 (0.76) 1.96 (0.72)	4.98 (1.06) 3.73 (0.93) *** 1.34 (0.36) 1.29 (0.32) * 2.79 (0.97) 1.92 (0.78) ***
Triglycerides (mmol/L)				
Baseline Week 4	2.01 (1.07) 0.98 (0.49)	1.96 (0.80) 1.06 (0.46)	1.74 (0.79) 1.07 (0.54)	1.91 (0.89) 1.04 (0.49) ***
Type 2 diabetes	7/20	7/20	7/20	21/60
Fasting glucose (mmol/L)				
Baseline Week 4	6.32 (1.80) 5.70 (1.46)	6.02 (1.70) 5.50 (1.39)	6.07 (1.85) 5.19 (0.77)	6.17 (1.77) 5.46 (1.23) ***
HbA1c (mmol/mol)				
Baseline Week 4	44.3 (11.5) 40.4 (8.3)	48.1 (12.9) 42.5 (8.6)	44.6 (11.5) 39.3 (7.4)	45.6 (11.9) 40.7 (8.1) ***
CRP (mg/L)				
Baseline Week 4	2.0 (0.5, 4.0) 1.0 (0.5, 4.0)	2.0 (1.0, 5.0) 0.8 (0.5, 3.0)	2.5 (0.5, 6.0) 1.5 (0.5, 3.0)	2.0 (0.5, 5.0) 1.0 (0.5, 3.0) **
Fibroscan^®^ CAP (dB/m)				
Baseline Week 4	307 (51) 267 (55)	299 (58) 246 (51)	296 (63) 258 (69)	301 (57) 257 (58) ***

Data are mean (SD), median (Q1, Q3) or number of patients. * *p* < 0.05, ** *p* < 0.01, *** *p* < 0.001 (paired *t*-test or Wilcoxon signed rank test).

## Appendix B

**Table A2:** Changes in characteristics at week 16, after 12 weeks of treatment following randomisation to metronidazole/inulin, placebo/inulin or placebo/placebo.

Clinical Characteristics	Group MI: Metronidazole-Inulin (*n* = 19)	Group PI: Placebo-Inulin (*n* = 19)	Group PP: Placebo-Placebo (*n* = 18)	*p*-Value Group MI vs. PP	*p*-Value Group PI vs. PP
Body mass index (kg/m^2^)	0.10 (1.20)	0.12 (1.80)	0.21 (1.36)	0.792	0.859
Weight (kg)	0.35 (3.38)	0.29 (5.02)	0.47 (4.10)	0.922	0.910
Waist:hip ratio	−0.00 (0.04)	−0.01 (0.04)	−0.00 (0.06)	0.893	0.604
Blood pressure (mm Hg)					
Systolic Diastolic	3.4 (11.3) −1.4 (10.2)	2.2 (16.7) 0.4 (12.0)	−7.2 (18.4) 0.5 (13.8)	0.043 0.641	0.117 0.981
ALT (U/L)	−19.6 (25.5)	−2.2 (16.7)	−0.2 (24.5)	**0.026**	0.856
AST (U/L)	−14 (−32, −7)	−3 (−22, 2)	−4 (−9, 5)	**0.006**	0.849
GGT (U/L)	13 (−2, 29)	26 (−1, 91)	17 (8, 62)	0.274	0.693
Bilirubin (µmol/L)	−1 (−3, 1)	−2 (−3, 0)	1 (−2, 2)	0.115	0.033
Albumin (g/L)	0.13 (1.81)	−1.17 (2.46)	−1.14 (2.57)	0.131	0.979
Ferritin (µg/L)	−56.3 (60.1)	−27.6 (63.5)	−1.3 (72.7)	0.031	0.291
Cholesterol (mmol/L)					
Total HDL LDL	1.09 (1.10) 0.16 (0.15) 2.28 (5.56)	1.32 (1.13) 0.20 (0.24) 0.83 (0.77)	0.91 (0.57) 0.13 (0.17) 0.55 (0.49)	0.560 0.638 0.181	0.208 0.344 0.246
Triglycerides (mmol/L)	0.52 (0.32)	0.50 (0.79)	0.56 (0.64)	0.831	0.814
Fasting glucose (mmol/L)	0.39 (1.32)	0.18 (0.70)	1.14 (2.05)	0.228	0.077
HbA1c (mmol/mol)	1.94 (4.25)	0 (5.79)	2.06 (6.02)	0.947	0.316
CRP (mg/L)	0.0 (−1.0, 0.75)	1.0 (0.0, 1.5)	0.0 (0.0, 1.0)	0.884	0.235
Fibroscan CAP (dB/m)	14.5 (65.6)	22.4 (71.6)	10.8 (42.6)	0.859	0.605

Data are mean (SD) or median (Q1, Q3).
