# Supplementary material for: Randomised Double-Blind Placebo-Controlled Trial of Inulin with Metronidazole in Non-Alcoholic Fatty Liver Disease (NAFLD)
Source: Nutrients. 2020 Mar 27;12(4):937. doi: 10.3390/nu12040937 (PMC7230525; doi:10.3390/nu12040937)
Supplement: Supplementary file 1 [file nutrients-12-00937-s001.pdf]

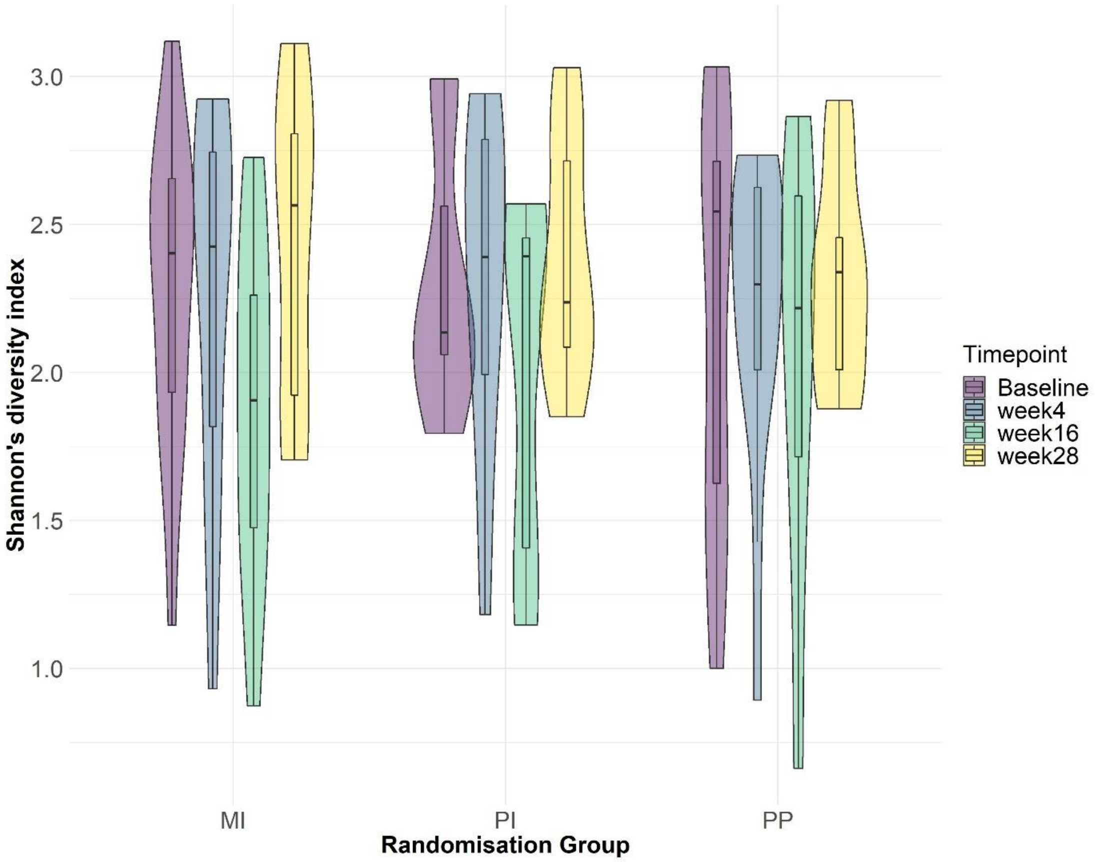

Figure S1. Violin plot on the Shannon's diversity index for each group on each time point. MI: metronidazole-inulin (Baseline, n=16; week4, n=11; week16, n=10; week28, n=8); PI: Placebo-inulin (Baseline, n=8; week4, n=12; week16, n=10; week28, n=10); PP: Placebo-placebo (Baseline, n=11; week4, n=15; week16, n=8; week28, n=8).

Table S1. DNA quality and quantity measured using a NanoPhotometer N60.

SubjectID	Treatment groups	Sample collection timeline							
		Baseline (pre VLCD)		Week 4 (after 4 weeks of VLCD)		Week 16 (12 weeks after randomisation)		Week 28 (follow-up visit)	
		DNA concentration (ng/uL)	260/280	DNA concentration	260/280	DNA concentration	260/280	DNA concentration	260/280
**1**	MI	42.400	1.941	22.300	1.806	-	-	-	-
2	MI	26.750	2.019	-	-	45.600	2.141	-	-
3	PI	-	-	-	-	-	-	40.400	1.985
4	PI	-	-	70.350	2.054	-	-	-	-
5	PI	-	-	-	-	-	-	119.750	1.970
7	PP	-	-	-	-	41.200	2.135	76.550	2.097
8	PI	-	-	-	-	-	-	82.900	2.022
9	PP	32.050	2.041	56.150	1.974	-	-	27.050	2.073
10	PP	12.800	1.910	11.450	1.893	37.500	1.963	28.500	1.875
11	MI	63.750	2.157	35.500	2.101	-	-	-	-
12	PP	20.200	1.780	34.450	1.852	-	-	-	-
13	MI	17.300	1.922	15.150	1.751	58.000	1.892	29.450	1.925
14	PI	45.400	1.924	25.200	1.806	46.800	1.906	-	-
15	PI	70.700	2.035	48.500	2.042	51.250	2.030	87.500	2.137
16	MI	45.400	1.944	24.750	2.124	58.300	1.908	30.900	1.974
18	PP	17.900	2.023	23.300	1.910	7.950	1.988	12.600	1.938
19	PP	-	-	57.050	1.991	-	-	-	-
20	PI	-	-	-	-	17.750	1.919	-	-
22	PP	-	-	38.250	1.848	-	-	-	-
23	MI	21.950	1.807	12.800	1.631	31.050	1.832	-	-
24	PP	35.000	2.128	27.500	2.060	73.900	2.209	16.450	1.982
26	PI	-	-	40.350	1.877	28.600	2.007	-	-
28	PI	-	-	40.050	2.147	-	-	-	-

29	PP	-	-	26.100	1.752	57.700	2.028	-	-
30	PI	33.400	1.931	52.500	2.043	33.250	1.950	23.200	1.834
31	PI	-	-	-	-	18.000	1.846	-	-
32	PP	12.250	2.042	29.850	1.913	39.750	1.771	-	-
33	MI	21.500	2.048	-	-	19.400	2.042	-	-
34	PP	58.150	2.158	56.750	2.034	69.800	1.986	52.350	2.021
35	PI	42.250	2.066	58.750	2.140	-	-	-	-
36	MI	16.750	2.055	63.250	1.802	20.750	2.173	-	-
37	MI	5.95	1.859	-	-	-	-	4.2500	2.125
38	PP	22.150	1.779	10.900	1.817	-	-	-	-
39	PI	-	-	-	-	-	-	8.050	2.403
40	PI	17.350	2.129	25.450	1.943	11.250	1.940	12.500	1.880
41	PI	-	-	-	-	4.800	1.600	-	-
42	PI	26.550	2.019	16.150	1.900	14.750	1.967	-	-
43	MI	12.400	2.016	27.950	1.908	31.550	1.966	16.950	1.982
44	PP	14.600	1.921	38.300	1.819	-	-	-	-
45	PP	39.700	2.129	48.100	2.167	-	-	-	-
46	MI	47.600	2.079	-	-	25.500	1.939	11.100	1.748
47	MI	51.000	2.094	4.0500	1.761	-	-	-	-
48	PP	12.200	2.122	12.400	1.865	18.950	2.191	43.650	2.030
49	MI	46.850	1.964	8.9000	1.854	-	-	23.800	2.125
50	PI	16.300	1.906	14.150	1.655	-	-	59.550	1.988
53	PI	54.450	2.110	25.550	1.886	-	-	29.900	1.980
54	MI	7.300	2.317	5.100	1.962	31.200	1.914	5.000	1.563
55	MI	18.100	2.129	7.8500	1.805	-	-	37.900	1.964
56	MI	65.000	1.937	-	-	32.550	1.955	43.750	1.971
58	PP	41.900	2.095	51.800	2.072	-	-	-	-
59	PP	-	-	-	-	59.350	1.982	59.750	1.924
60	PI	-	-	27.000	2.015	53.500	2.054	23.750	2.039

Table S2. Linear modeling results showing genera associated with the VLCD treatment.

Feature	Value	Coefficient	N	N.not.0	P.value	Q.value
k__Bacteria_p__Firmicutes_c__Clostridia_o__Clostridiales_f__Lachnospiraceae_g__Roseburia	week4	-0.11757	73	67	2.37E-06	0.000505
k__Bacteria_p__Firmicutes_c__Bacilli_o__Lactobacillales_f__Streptococcaceae_g__Streptococcus	week4	-0.02018	73	56	4.76E-06	0.000507
k__Bacteria_p__Firmicutes_c__Clostridia_o__Clostridiales_f__Veillonellaceae_g__Dialister	week4	-0.05847	73	40	0.000457	0.032459
k__Bacteria_p__Firmicutes_c__Clostridia_o__Clostridiales_f__Christensenellaceae_g__	week4	0.01761	73	41	0.004863	0.226181
k__Bacteria_p__Firmicutes_c__Clostridia_o__Clostridiales_f__Lachnospiraceae___	week4	0.01911	73	64	0.005309	0.226181
k__Bacteria_p__Firmicutes_c__Clostridia_o__Clostridiales_f__Dehalobacteriaceae_g__Dehalobacterium	week4	0.006089	73	26	0.010986	0.353771
k__Bacteria_p__Bacteroidetes_c__Bacteroidia_o__Bacteroidales_f__Bacteroidaceae_g__Bacteroides	week4	0.13123	73	73	0.017087	0.363955
k__Bacteria_p__Firmicutes_c__Clostridia_o__Clostridiales_f__Ruminococcaceae_g__Anaerotruncus	week4	0.006331	73	44	0.021915	0.394094
k__Bacteria_p__Actinobacteria_c__Coriobacteriia_o__Coriobacteriales_f__Coriobacteriaceae_g__Collinsella	week4	-0.02022	73	68	0.031263	0.512226
k__Bacteria_p__Proteobacteria_c__Deltaproteobacteria_o__Desulfovibrionales_f__Desulfovibrionaceae_g__Desulfovibrio	week4	0.014862	73	27	0.035998	0.547683
k__Bacteria_p__Firmicutes_c__Clostridia_o__Clostridiales_f__Ruminococcaceae_g__Gemmiger	week4	-0.02177	73	65	0.043413	0.601146
k__Bacteria_p__Firmicutes_c__Clostridia_o__Clostridiales_f__Veillonellaceae_g__Veillonella	week4	-0.00925	73	40	0.045156	0.601146
k__Bacteria_p__Bacteroidetes_c__Bacteroidia_o__Bacteroidales_f___Odoribacteraceae__g__Odoribacter	week4	0.014542	73	51	0.06343	0.711088
k__Bacteria_p__Proteobacteria_c__Gammaproteobacteria_o__Enterobacteriales_f__Enterobacteriaceae_g__Enterobacter	week4	0.003923	73	28	0.085399	0.826816

k__Bacteria_p__Bacteroidetes_c__Bacteroidia_o__Bacteroidales_f__Porphyromonadaceae_g__Parabacteroides	week4	0.023817	73	70	0.149417	1
k__Bacteria_p__Firmicutes_c__Clostridia_o__Clostridiales_f__Lachnospiraceae_g__Blautia	week4	0.015649	73	71	0.173858	1
k__Bacteria_p__Firmicutes_c__Clostridia_o__Clostridiales_f__Lachnospiraceae_g___Ruminococcus_	week4	0.011154	73	65	0.313664	1
k__Bacteria_p__Bacteroidetes_c__Bacteroidia_o__Bacteroidales_f___Barnesiellaceae____	week4	-0.00508	73	16	0.319608	1
k__Bacteria_p__Firmicutes_c__Erysipelotrichi_o__Erysipelotrichales_f__Erysipelotrichaceae_g___Eubacterium_	week4	0.004704	73	52	0.395803	1

Table S3. Linear modeling results showing genera associated with the antibiotic/prebiotic treatment period.

Feature	Value	Coefficient	N	N.not.0	P.value	Q.value
k__Bacteria_p__Firmicutes_c__Clostridia_o__Clostridiales_f__Lachnospiraceae_g__Roseburia	week4	-0.078173	66	59	0.000166	0.026219
k__Bacteria_p__Firmicutes_c__Clostridia_o__Clostridiales_f__Ruminococcaceae_g__Anaerotruncus	week4	0.0098941	66	41	0.000356	0.026219
k__Bacteria_p__Firmicutes_c__Clostridia_o__Clostridiales_f__Lachnospiraceae___	week4	0.0304695	66	59	0.000386	0.026219
k__Bacteria_p__Firmicutes_c__Clostridia_o__Clostridiales_f__Lachnospiraceae_g__Blautia	week4	0.0378814	66	62	0.005721	0.211808
k__Bacteria_p__Proteobacteria_c__Deltaproteobacteria_o__Desulfovibrionales_f__Desulfovibrionaceae_g__Desulfovibrio	week4	0.0290155	66	21	0.006056	0.211808
k__Bacteria_p__Firmicutes_c__Clostridia_o__Clostridiales_f__Ruminococcaceae___	week4	0.0306268	66	61	0.00623	0.211808
k__Bacteria_p__Firmicutes_c__Clostridia_o__Clostridiales_f__Lachnospiraceae_g__Dorea	week4	0.023113	66	65	0.012616	0.321714
k__Bacteria_p__Firmicutes_c__Clostridia_o__Clostridiales_f__Ruminococcaceae_g__Oscillospira	week4	0.0534824	66	64	0.020661	0.421491
k__Bacteria_p__Firmicutes_c__Clostridia_o__Clostridiales_f__Lachnospiraceae_g___Ruminococcus_	week4	0.0226583	66	56	0.023205	0.430353
k__Bacteria_p__Firmicutes_c__Clostridia_o__Clostridiales_f__Christensenellaceae_g__	week4	0.0175659	66	39	0.033743	0.488615
k__Bacteria_p__Firmicutes_c__Clostridia_o__Clostridiales_f__Dehalobacteriaceae_g__Dehalobacterium	week4	0.0063093	66	23	0.036038	0.488615
k__Bacteria_p__Firmicutes_c__Clostridia_o__Clostridiales_f__Ruminococcaceae_g__Ruminococcus	week4	0.030421	66	64	0.038323	0.488615
k__Bacteria_p__Proteobacteria_c__Gammaproteobacteria_o__Enterobacteriales_f__Enterobacteriaceae_g__Enterobacter	week4	0.0598402	66	21	0.041987	0.503844
k__Bacteria_p__Bacteroidetes_c__Bacteroidia_o__Bacteroidales_f__Porphyromonadaceae_g__Parabacteroides	week4	0.0326368	66	64	0.051884	0.588018
k__Bacteria_p__Firmicutes_c__Clostridia_o__Clostridiales_f__Lachnospiraceae_g__	week4	0.0282031	66	65	0.072873	0.607803
k__Bacteria_p__Bacteroidetes_c__Bacteroidia_o__Bacteroidales_f___Odoribacteraceae__g__Odoribacter	week4	0.0174361	66	46	0.086403	0.607803

k__Bacteria_p__Firmicutes_c__Clostridia_o__Clostridiales_f___g__	week4	0.025575	66	59	0.163102	0.811533
k__Bacteria_p__Actinobacteria_c__Actinobacteria_o__Bifidobacteriales_f__Bifidobacteriaceae_g__Bifidobacterium	week4	-0.0145479	66	50	0.190028	0.88104
k__Bacteria_p__Firmicutes_c__Erysipelotrichi_o__Erysipelotrichales_f__Erysipelotrichaceae_g___Eubacterium_	week4	0.0080958	66	47	0.202493	0.91797
k__Bacteria_p__Firmicutes_c__Erysipelotrichi_o__Erysipelotrichales_f__Erysipelotrichaceae_g__Clostridium	week4	0.002867	66	28	0.293843	0.952471
k__Bacteria_p__Bacteroidetes_c__Bacteroidia_o__Bacteroidales_f__Rikenellaceae_g__Alistipes	week4	0.0069049	66	54	0.377679	1
k__Bacteria_p__Bacteroidetes_c__Bacteroidia_o__Bacteroidales_f__Prevotellaceae_g__Prevotella	week4	-0.0184709	66	34	0.443291	1
k__Bacteria_p__Proteobacteria_c__Deltaproteobacteria_o__Desulfovibrionales_f__Desulfovibrionaceae_g__	week4	0.0022412	66	26	0.444285	1
k__Bacteria_p__Firmicutes_c__Erysipelotrichi_o__Erysipelotrichales_f__Erysipelotrichaceae_g__Coprobacillus	week4	-0.0011672	66	16	0.769155	1
k__Bacteria_p__Bacteroidetes_c__Bacteroidia_o__Bacteroidales_f__Bacteroidaceae_g__Bacteroides	week4	0.0095178	66	65	0.840294	1

Table S4. Linear modeling results showing suggestive association between genus *Turicibacter* and plasma ALT levels.

Feature	Value	Coefficient	N	N.not.0	P.value	Q.value
k__Bacteria_p__Firmicutes_c__Bacilli_o__Turicibacterales_f__Turicibacteraceae_g__Turicibacter	ALT	0.000315	82	21	0.00066	0.085764
k__Bacteria_p__Firmicutes_c__Clostridia_o__Clostridiales_f__Lachnospiraceae_g__	ALT	0.000899	82	82	0.002311	0.150211
k__Bacteria_p__Firmicutes_c__Clostridia_o__Clostridiales_f__Veillonellaceae_g__Veillonella	ALT	0.000265	82	47	0.046431	0.660741
k__Bacteria_p__Firmicutes_c__Clostridia_o__Clostridiales______	ALT	0.000283	82	77	0.049201	0.660741
k__Bacteria_p__Bacteroidetes_c__Bacteroidia_o__Bacteroidales_f__Bacteroidaceae_g__Bacteroides	ALT	-0.00266	82	82	0.061736	0.666999
k__Bacteria_p__Firmicutes_c__Clostridia_o__Clostridiales_f__Lachnospiraceae_g__Lachnospira	ALT	0.000496	82	70	0.071831	0.666999
k__Bacteria_p__Firmicutes_c__Bacilli_o__Lactobacillales_f__Streptococcaceae_g__Streptococcus	ALT	0.00019	82	64	0.09056	0.735799
k__Bacteria_p__Firmicutes_c__Clostridia_o__Clostridiales_f__Lachnospiraceae_g__Coprococcus	ALT	0.000425	82	78	0.130709	0.917352
k__Bacteria_p__Firmicutes_c__Clostridia_o__Clostridiales_f__Lachnospiraceae_g__Roseburia	ALT	0.000679	82	80	0.149861	0.917352
k__Bacteria_p__Verrucomicrobia_c__Verrucomicrobiae_o__Verrucomicrobiales_f__Verrucomicrobiaceae_g__Akkermansia	ALT	-0.00068	82	43	0.151329	0.917352
k__Bacteria_p__Actinobacteria_c__Coriobacteriia_o__Coriobacteriales_f__Coriobacteriaceae_g__Slackia	ALT	7.91E-05	82	30	0.183142	0.923009
k__Bacteria_p__Bacteroidetes_c__Bacteroidia_o__Bacteroidales_f___Barnesiellaceae__g__Barnesiella	ALT	0.000186	82	28	0.214274	0.923009
k__Bacteria_p__Firmicutes_c__Bacilli_o__Lactobacillales_f__Lactobacillaceae_g__Lactobacillus	ALT	0.000246	82	28	0.261751	0.923009
k__Bacteria_p__Firmicutes_c__Clostridia_o__Clostridiales_f__Lachnospiraceae___	ALT	-0.00015	82	75	0.267555	0.923009

k__Bacteria_p__Bacteroidetes_c__Bacteroidia_o__Bacteroidales_f___Odoribacteraceae__g__Odoribacter	ALT	0.000256	82	62	0.268442	0.923009
k__Bacteria_p__Bacteroidetes_c__Bacteroidia_o__Bacteroidales_f___Paraprevotellaceae__g__Paraprevotella	ALT	0.000216	82	26	0.281156	0.923009
k__Bacteria_p__Bacteroidetes_c__Bacteroidia_o__Bacteroidales_f__Porphyromonadaceae_g__Parabacteroides	ALT	-0.00039	82	81	0.289145	0.923009
k__Bacteria_p__Firmicutes_c__Clostridia_o__Clostridiales_f__Lachnospiraceae_g__Ruminococcus	ALT	-0.00013	82	28	0.289975	0.923009
k__Bacteria_p__Firmicutes_c__Erysipelotrichi_o__Erysipelotrichales_f__Erysipelotrichaceae_g__	ALT	0.000201	82	62	0.29611	0.923009
k__Bacteria_p__Firmicutes_c__Clostridia_o__Clostridiales_f__Lachnospiraceae_g___Ruminococcus_	ALT	0.000223	82	70	0.326751	0.923009
k__Bacteria_p__Firmicutes_c__Erysipelotrichi_o__Erysipelotrichales_f__Erysipelotrichaceae_g__Clostridium	ALT	6.34E-05	82	31	0.350991	0.923009
k__Bacteria_p__Firmicutes_c__Clostridia_o__Clostridiales_f__Ruminococcaceae_g__Oscillospira	ALT	-0.00047	82	81	0.365377	0.923009
k__Bacteria_p__Firmicutes_c__Clostridia_o__Clostridiales_f___g__	ALT	-0.00019	82	73	0.397243	0.923009
k__Bacteria_p__Firmicutes_c__Clostridia_o__Clostridiales_f__Clostridiaceae_g__Clostridium	ALT	8.73E-05	82	38	0.398325	0.923009
k__Bacteria_p__Firmicutes_c__Clostridia_o__Clostridiales_f__Ruminococcaceae_g__	ALT	-0.0004	82	77	0.408791	0.923009
k__Bacteria_p__Firmicutes_c__Clostridia_o__Clostridiales_f__Ruminococcaceae_g__Anaerotruncus	ALT	-4.62E-05	82	43	0.410558	0.923009
k__Bacteria_p__Firmicutes_c__Clostridia_o__Clostridiales_f__Lachnospiraceae_g__Anaerostipes	ALT	7.46E-05	82	50	0.422095	0.923009
k__Bacteria_p__Proteobacteria_c__Deltaproteobacteria_o__Desulfovibrionales_f__Desulfovibrionaceae_g__Desulfovibrio	ALT	-0.00016	82	35	0.432009	0.923009
k__Bacteria_p__Proteobacteria_c__Betaproteobacteria_o__Burkholderiales_f__Alcaligenaceae_g__Sutterella	ALT	0.000289	82	76	0.438002	0.923009
k__Bacteria_p__Actinobacteria_c__Actinobacteria_o__Bifidobacteriales_f__Bifidobacteriaceae_g__Bifidobacterium	ALT	0.00021	82	66	0.484871	0.923009

k__Bacteria_p__Firmicutes_c__Clostridia_o__Clostridiales_f__Lachnospiraceae_g__Blautia	ALT	0.000167	82	81	0.490913	0.923009
k__Bacteria_p__Firmicutes_c__Clostridia_o__Clostridiales_f__Lachnospiraceae_g__Dorea	ALT	-0.00015	82	78	0.4915	0.923009
k__Bacteria_p__TM7_c__TM7_3_o___f___g__	ALT	1.84E-05	82	26	0.514072	0.92981
k__Bacteria_p__Firmicutes_c__Clostridia_o__Clostridiales_f__Ruminococcaceae_g__Gemmiger	ALT	0.000177	82	73	0.525466	0.92981
k__Bacteria_p__Proteobacteria_c__Gammaproteobacteria_o__Enterobacteriales_f__Enterobacteriaceae___	ALT	-7.12E-05	82	37	0.555149	0.937178
k__Bacteria_p__Firmicutes_c__Clostridia_o__Clostridiales_f__Peptostreptococcaceae___	ALT	3.66E-05	82	26	0.560943	0.937178
k__Bacteria_p__Proteobacteria_c__Deltaproteobacteria_o__Desulfovibrionales_f__Desulfovibrionaceae_g__Bilophila	ALT	-0.00011	82	57	0.567322	0.937178
k__Bacteria_p__Actinobacteria_c__Coriobacteriia_o__Coriobacteriales_f__Coriobacteriaceae_g__Eggerthella	ALT	-2.52E-05	82	23	0.56851	0.937178
k__Bacteria_p__Firmicutes_c__Clostridia_o__Clostridiales_f__Veillonellaceae_g__Phascolarctobacterium	ALT	-0.00021	82	50	0.569516	0.937178
k__Bacteria_p__Proteobacteria_c__Deltaproteobacteria_o__Desulfovibrionales_f__Desulfovibrionaceae_g__	ALT	3.56E-05	82	27	0.580933	0.944016
k__Bacteria_p__Bacteroidetes_c__Bacteroidia_o__Bacteroidales_f__Prevotellaceae_g__Prevotella	ALT	0.000383	82	48	0.657798	1
k__Bacteria_p__Firmicutes_c__Clostridia_o__Clostridiales_f__Ruminococcaceae_g__Butyricicoccus	ALT	5.27E-05	82	48	0.66577	1
k__Bacteria_p__Firmicutes_c__Clostridia_o__Clostridiales_f__Lachnospiraceae_g__Clostridium	ALT	-7.19E-05	82	76	0.667884	1
k__Bacteria_p__Proteobacteria_c__Gammaproteobacteria_o__Enterobacteriales_f__Enterobacteriaceae_g__Enterobacter	ALT	0.000212	82	26	0.693077	1
k__Bacteria_p__Firmicutes_c__Clostridia_o__Clostridiales_f__Ruminococcaceae___	ALT	-7.85E-05	82	76	0.711964	1
k__Bacteria_p__Firmicutes_c__Erysipelotrichi_o__Erysipelotrichales_f__Erysipelotrichaceae_g___Eubacterium_	ALT	-5.64E-05	82	61	0.713943	1

k__Bacteria_p__Firmicutes_c__Clostridia_o__Clostridiales_f___Mogibacteriaceae__g__	ALT	3.71E-05	82	65	0.749266	1
k__Bacteria_p__Bacteroidetes_c__Bacteroidia_o__Bacteroidales_f___Barnesiellaceae__g__	ALT	-5.48E-05	82	33	0.756386	1
k__Bacteria_p__Proteobacteria_c__Gammaproteobacteria_o__Enterobacteriales_f__Enterobacteriaceae_g__Escherichia	ALT	-8.11E-05	82	58	0.761481	1
k__Bacteria_p__Firmicutes_c__Erysipelotrichi_o__Erysipelotrichales_f__Erysipelotrichaceae_g__Holdemania	ALT	-1.69E-05	82	42	0.777823	1
k__Bacteria_p__Bacteroidetes_c__Bacteroidia_o__Bacteroidales_f__Rikenellaceae_g__	ALT	1.95E-05	82	24	0.815435	1
k__Bacteria_p__Bacteroidetes_c__Bacteroidia_o__Bacteroidales_f__Rikenellaceae_g__Alistipes	ALT	-3.48E-05	82	69	0.829132	1
k__Bacteria_p__Bacteroidetes_c__Bacteroidia_o__Bacteroidales_f___Odoribacteraceae__g__Butyricimonas	ALT	-1.67E-05	82	21	0.850824	1
k__Bacteria_p__Firmicutes_c__Clostridia_o__Clostridiales_f__Ruminococcaceae_g__Ruminococcus	ALT	-5.78E-05	82	82	0.861137	1
k__Bacteria_p__Firmicutes_c__Clostridia_o__Clostridiales_f__Veillonellaceae_g__Acidaminococcus	ALT	-3.33E-05	82	25	0.865238	1
k__Bacteria_p__Actinobacteria_c__Coriobacteriia_o__Coriobacteriales_f__Coriobacteriaceae_g__	ALT	-1.19E-05	82	62	0.916727	1
k__Bacteria_p__Firmicutes_c__Clostridia_o__Clostridiales_f__Veillonellaceae_g__Dialister	ALT	-5.24E-05	82	46	0.932128	1
k__Bacteria_p__Actinobacteria_c__Coriobacteriia_o__Coriobacteriales_f__Coriobacteriaceae_g__Collinsella	ALT	-2.30E-05	82	75	0.934909	1
k__Bacteria_p__Firmicutes_c__Clostridia_o__Clostridiales_f__Ruminococcaceae_g__Faecalibacterium	ALT	3.10E-05	82	76	0.935072	1
k__Bacteria_p__Firmicutes_c__Clostridia_o__Clostridiales_f__Christensenellaceae_g__	ALT	5.61E-06	82	43	0.963799	1
k__Bacteria_p__Firmicutes_c__Clostridia_o__Clostridiales_f__Veillonellaceae_g__Megasphaera	ALT	-9.85E-06	82	27	0.983083	1
k__Bacteria_p__Firmicutes_c__Erysipelotrichi_o__Erysipelotrichales_f__Erysipelotrichaceae_g__Catenibacterium	ALT	-2.87E-06	82	27	0.984265	1
